# The Effect of Negative and Positive Emotionality on Associative Memory: An fMRI Study

**DOI:** 10.1371/journal.pone.0024862

**Published:** 2011-09-14

**Authors:** Go Okada, Yasumasa Okamoto, Yoshihiko Kunisato, Shiori Aoyama, Yoshiko Nishiyama, Shinpei Yoshimura, Keiichi Onoda, Shigeru Toki, Hidehisa Yamashita, Shigeto Yamawaki

**Affiliations:** 1 Department of Psychiatry and Neurosciences, Division of Frontier Medical Science, Programs for Biomedical Research, Graduate School of Biomedical Sciences, Hiroshima University, Hiroshima, Japan; 2 Core Research for Evolutional Science and Technology, Japan Science and Technology Corporation, Kawaguchi, Japan; 3 Research Fellow of the Japan Society for the Promotion of Science, Tokyo, Japan; 4 Department of Neurology, Shimane University, Izumo, Japan; The University of Melbourne, Australia

## Abstract

In general, emotion is known to enhance memory processes. However, the effect of emotion on associative memory and the underling neural mechanisms remains largely unexplored. In this study, we explored brain activation during an associative memory task that involved the encoding and retrieval of word and face pairs. The word and face pairs consisted of either negative or positive words with neutral faces. Significant hippocampal activation was observed during both encoding and retrieval, regardless of whether the word was negative or positive. Negative and positive emotionality differentially affected the hemodynamic responses to encoding and retrieval in the amygdala, with increased responses during encoding negative word and face pairs. Furthermore, activation of the amygdala during encoding of negative word and neutral face pairs was inversely correlated with subsequent memory retrieval. These findings suggest that activation of the amygdala induced by negative emotion during encoding may disrupt associative memory performance.

## Introduction

The ability to learn and remember new associations between previously unrelated information is an important aspect of declarative memory. Declarative memory is associative, linking together component parts, such as words and objects, either directly or via spatial, temporal or other relationships. Previous neuroimaging studies have provided crucial information concerning the neural correlates that underlie this process. A variety of associative encoding tasks results in robust hippocampal activation, including the encoding of word pairs [1], [2], [3], [4] and triplets [5], [6], object pairs [7], and name–face pairs [8], [9], [10].

On the other hand, the relationship between memory and emotion is of paramount importance, given that people experience various affective states over the course of daily life. Although a recent review on memory and emotion has demonstrated that emotion may enhance memory processes that occur at all stages, including encoding, storage, and retrieval [11], we previously reported that negative emotionality does not necessarily promote good memory performance and associated hippocampal activation [12]. This discrepancy may be due to procedural differences. The most likely explanation is that the encoding of an association between items may have played a key role. The possible effects of emotionality associated with memory for paired items is unclear, even though the medial temporal lobe (including the hippocampus) showed greater activation for emotional items than for neutral items during both encoding [13] and retrieval [14] in studies of memory for single items. Furthermore, although much functional neuroimaging evidence has linked the memory-enhancing effect of emotion to amygdalic modulation during encoding [13], [15], [16], [17], [18], [19], [20] and retrieval [14], whether similar emotionality effects can be observed on associative memory remains unclear. Indeed, emotion does not necessarily enhance memory. When faced with negative events, people tend to pay attention to central features of such events while ignoring peripheral details [21], [22]. As a result, memory of the negative event itself is enhanced, whereas memory of peripheral events is impaired. Furthermore, the difference between memory for gist and memory for detail can be more pronounced for negative than for positive events [23].

In this study, we hypothesized that negative emotion does not necessarily promote good associative memory performance, and that the amygdala has disparate influences on associative memory for positive and negative information. We used fMRI to investigate the effect of emotional (negative or positive) item valence on brain activation during an associative memory task, and examined the relationship between memory performance and brain activation affected by emotion during encoding and retrieval.

## Results

### Behavioral results

**Figure 1:**
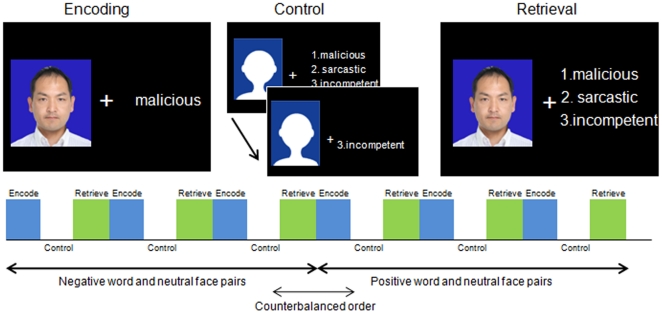
Face-Word Association Paradigm. Subjects were asked to learn pairs of neutral face and emotional (positive or negative) words related to personality-trait by pressing a button. After the control task in which subjects were asked to press one of the target button, each face was shown with 3 words and subjects were asked to indicate, via button press, which word was previously paired with that face. (The picture of one of the authors was used in the Figure instead of that from the database of SOFTPIA JAPAN to protect the privacy of subjects participated in the database).

During the fMRI protocol, 15 healthy volunteers performed a novel face-emotional word paired associate task consisted of ‘encoding’, in which subjects were asked to remember pairs of neutral face and emotional (positive or negative) words, and ‘control’ and ‘retrieval’, in which subjects were asked to indicate the word that was previously paired with that face (Fig. 1; see Methods for details). The mean correct response rates (mean ± SD) during retrieval were 48.9±14.7% for negative word and neutral face pairs and 58.1±13.3% for positive word and neutral face pairs. Accuracy rates across the two emotional conditions differed significantly (paired t-test, *t* = −2.208, *p* = 0.044).

### Group analysis on each contrast

We performed fMRI group analysis on the four contrasts, subtracting the control condition from each experimental condition, according to a random effect model. All activations satisfying our criteria for significance are shown in Tables 1 and 2. We observed significant activation of the hippocampus during all of the 4 conditions, and significant activation of the left amygdala only during the negative encoding condition.

**Table 1:** Results of one sample t-test for negative word and neutral face pairs.

Region	BA	Side	peak level		cluster level		x	y	z
			Z	p	k_E_	p			
Encoding									
Cerebellum		R	5.50	0.002	25	0.002	42	−52	−30
Inferior Frontal Gyrus	47	L	5.21	0.007	30	0.001	−42	−18	−10
Hippocampus		R	3.97	0.008^a^	71^a^	0.005^a^	28	−38	0
Hippocampus		L	3.37	0.050^a^	1^a^	0.044^a^	−30	−22	−16
Amygdala		L	2.91	0.050^b^	1^b^	0.046^b^	−30	0	−22
Retrieval									
Inferior Frontal Gyrus	47	L	5.70	0.001	132	0.000	−34	18	−6
Cerebellum		R	5.44	0.003	89	0.000	44	−54	−28
Precentral Gyrus	9	L	5.42	0.003	57	0.000	−38	4	38
Lingual Gyrus	18	L	5.34	0.004	451	0.000	−10	−82	−14
Cerebellum		L	5.29	0.005	92	0.000	0	−60	−44
Medial Frontal Gyrus	32	L	5.14	0.009	65	0.000	−8	12	50
Angular Gyrus	39	L	5.13	0.009	76	0.000	−32	−62	38
Cuneus	17	L	4.92	0.021	8	0.010	−24	−80	12
Middle Occipital Gyrus	19	L	4.83	0.030	19	0.003	−26	−92	4
Cerebellum		R	4.82	0.031	11	0.006	8	−74	−50
Superior Temporal Gyrus	38	L	4.76	0.036	4	0.018	−46	16	−16
Inferior Occipital Gyrus	18	L	4.74	0.042	5	0.015	−34	−88	−10
Cuneus	17	R	4.73	0.044	2	0.026	20	−96	−10
Hippocampus		L	3.65	0.023^a^	14^a^	0.023^a^	−24	−30	−6

BA, Brodmann area; L, Left; R, Right; Z, Z value of the peak activation within the cluster; Coordinates for the peak voxel are listed as MNI coordinates. p, corrected p value for whole brain or region of interest (^a^ bilateral hippocampus which include 1667 voxels or ^b^ bilateral amygdala which include 306 voxels); k_E_, cluster size (voxels) difined by the same peak-level FWE thresholds and used for the cluster level testing.

**Table 2:** Results of one sample t-test for positive word and neutral face pairs.

Region	BA	Side	peak level		cluster level		x	y	z
			Z	p	k_E_	p			
Encoding									
Cerebellum		R	4.85	0.031	5	0.013	42	−52	−30
Cerebellum		L	4.72	0.048	1	0.032	−6	−50	−40
Hippocampus		R	4.45	0.002^a^	103^a^	0.002^a^	36	−36	−6
Hippocampus		R	4.04	0.007^a^	4^a^	0.035^a^	40	−16	−22
Hippocampus		L	3.54	0.033^a^	13^a^	0.022^a^	−32	−22	−18
Hippocampus		L	3.49	0.038^a^	1^a^	0.043^a^	−26	−40	4
Retrieval									
Cerebellum		R	5.91	0.000	174	0.000	42	−54	−30
Middle Occipital Gyrus	18	L	5.89	0.000	877	0.000	−30	−34	−14
Insula	13	R	5.73	0.001	121	0.000	34	24	6
Precuneus	7	L	5.61	0.002	130	0.000	−28	−72	38
Medial Frontal Gyrus	6	L	5.46	0.003	234	0.000	−6	14	50
Inferior Frontal Gyrus	47	L	5.22	0.009	45	0.000	−34	18	−4
Inferior Occipital Gyrus	18	R	5.19	0.010	71	0.000	22	−92	−12
Cerebellum		R	5.10	0.014	8	0.006	10	−74	−34
Inferior Frontal Gyrus	9	L	5.05	0.017	128	0.000	−38	4	34
Fusiform Gyrus	19	R	4.98	0.023	13	0.002	32	−80	−20
Cerebellum		R	4.97	0.023	13	0.002	2	−62	−42
Cerebellum		R	4.94	0.026	11	0.003	36	−72	−28
Cuneus	18	L	4.88	0.033	5	0.010	−24	−82	12
Caudate		L	4.86	0.035	11	0.003	−12	−8	18
Inferior Frontal Gyrus		L	4.84	0.038	5	0.010	−46	16	2
Hippocampus		L	4.40	0.002^a^	114^a^	0.001^a^	−24	−30	−4
Hippocampus		R	4.12	0.006^a^	151^a^	0.000^a^	24	−26	−8

BA, Brodmann area; L, Left; R, Right; Z, Z value of the peak activation within the cluster; Coordinates for the peak voxel are listed as MNI coordinates. p, corrected p value for whole brain or region of interest (^a^ bilateral hippocampus which include 1667 voxels or ^b^ bilateral amygdala which include 306 voxels); k_E_, cluster size (voxels) difined by the same peak-level FWE thresholds and used for cluster level testing.

### Correlation between activations in regions detected by one sample t-tests and associative memory performances

We conducted a secondary correlation analysis to examine the relationship between brain activation in regions detected by one sample t-tests and associative memory performance. There wasn't positive correlation between brain activation and associative memory performance in any areas. In contrast, this analysis revealed that left amygdala and hippocampus activation during encoding of negative word and neutral face pairs (contrast estimate of ‘negative encoding – control’) was inversely correlated (*r* = −0.527, *p* = 0.043, and *r* = −0.519, *p* = 0.047, respectively) with successful retrieval (Table 3).

**Table 3:** Correlation between activations in regions detected by one sample t-tests and associative memory performances.

	Region (peak coordinate)	Correct respons rate (negative)	Correct respons rate (positive)
Encoding (negative)	Hippocampus (28 −38 0)	*r* = −0.446, *p* = 0.096	
	Hippocampus (−30 −22 −16)	*r* = −0.527, *p* = 0.043*	
	Amygdala (−30 −0 −22)	*r* = −0.519, *p* = 0.047*	
Retrieval (negative)	Hippocampus (−24 −30 −6)	*r* = 0.037, *p* = 0.895	
Encoding (positive)	Hippocampus (36 −36 −6)		*r* = 0.121, *p* = 0.668
	Hippocampus (40 −16 −22)		*r* = 0.130, *p* = 0.643
	Hippocampus (−32 −22 −18)		*r* = −0.077, *p* = 0.786
	Hippocampus (−26 −40 4)		*r* = −0.000, *p* = 0.999
Retrieval (positive)	Hippocampus (−24 −30 −4)		*r* = −0.140, *p* = 0.618
	Hippocampus (24 −26 −8)		*r* = 0.079, *p* = 0.780

*r*, correlation coefficiency; *p*, p-value;

*, *p*<0.05.

### Differential effects of negative and positive emotion on encoding and retrieval

**Figure 2:**
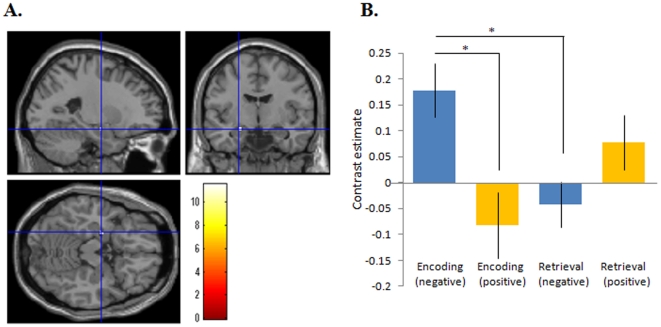
Differential effects of negative and positive emotion on encoding and retrieval. A. shows the brain region in which task×emotion interaction was detected (MNI coordinate: x = −24, y = −8, z = −16). B. shows the graph displaying the contrast estimates (mean ± SE) for the region of interest shown in Figure 2A for the 4 contrasts (negative encoding, positive encoding, negative retrieval, and positive retrieval compared to the relevant control) *: *p*<0.05.

A 2×2 ANOVA was performed to examine the differential effects of negative and positive emotion on encoding and retrieval. All activations satisfying our criteria for significance are shown in Table 4. This analysis revealed significant emotion×task interactions in the left amygdala (Fig. 2A). Post hoc analysis (corrected by Bonferroni) of the averages of contrast estimates of voxels in this cluster revealed that the ‘Encoding of negative word and neutral face pairs’ showed a greater BOLD response compared with the ‘Encoding of positive word and neutral face pairs’ (*F* = 7.761, *p* = 0.012) and the ‘Retrieval of negative word and neutral face pairs’ (*F* = 8.335, *p* = 0.015) in this area (Fig. 2B).

**Table 4:** Results of 2×2 ANOVA.

Region	BA	Side	peak level		cluster level	x	y	z
			Z	p	k_E_			
Main effect of task								
Encoding>Retrieval								
Angular Gyrus	39	L	6.46	0.000	312	−56	−62	38
Parahippocampal Gyrus	37	R	4.94	0.007	29	40	−38	−20
Superior Frontal Gyrus	9	L	4.71	0.020	7	−20	44	44
Superior Frontal Gyrus	6	L	4.56	0.038	2	−16	14	60
Middle Temporal Gyrus	39	L	4.53	0.042	2	−40	−66	16
Hippocampus		R	4.86	0.000^a^	112^a^	38	−18	−20
Hippocampus		L	3.56	0.024^a^	6^a^	−32	−26	−16
Retrieval>Encoding								
Inferior Occipital Gyrus	17	L	>8	0.000	13337	−10	−92	−12
Insula	13	R	5.72	0.000	217	32	26	2
Midbrain		L	5.63	0.000	500	−4	−20	−6
Inferior Frontal Gyrus	47	L	5.41	0.001	164	−30	22	−4
Inferior Frontal Gyrus	9	L	5.31	0.001	53	−58	4	30
Hippocampus		L	4.12	0.003^a^	8^a^	−20	−30	−4
Main effect of emotion								
No area								
Interaction Task×Emotion								
Amygdala		L	3.02	0.036^b^	6^b^	−24	−8	−16

BA, Brodmann area; L, Left; R, Right; Z, Z value of the peak activation within the cluster; Coordinates for the peak voxel are listed as MNI coordinates. p, corrected p value for whole brain or region of interest (^a^ bilateral hippocampus which include 1667 voxels or ^b^ bilateral amygdala which include 306 voxels); k_E_, cluster size (voxels) difined by the same peak-level FWE thresholds.

### Correlation analysis between amygdala activation and associative memory performance

**Figure 3:**
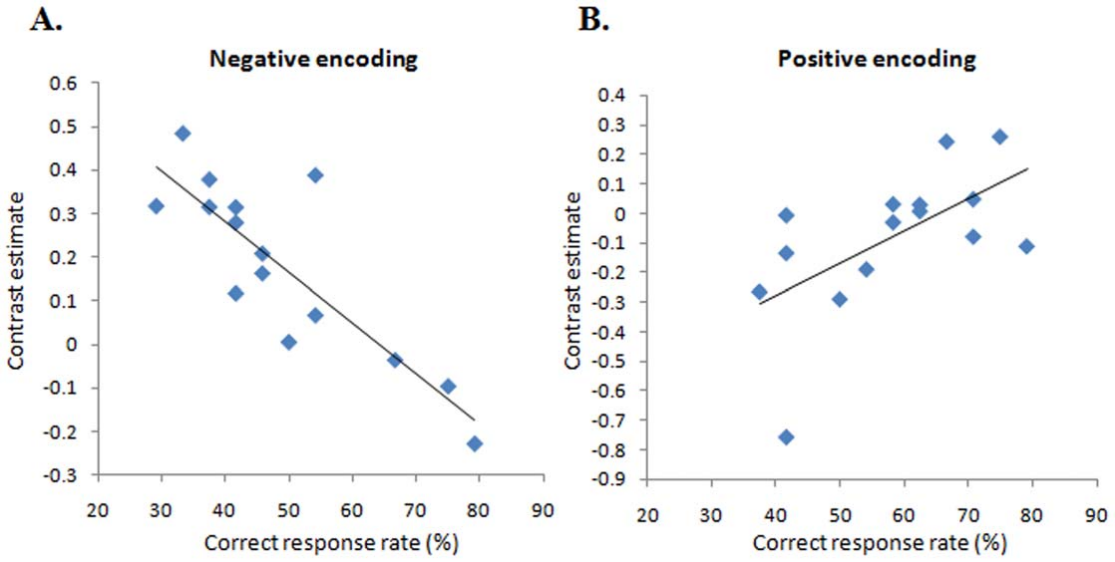
Correlation between amygdala activation and associative memory performance. A. shows the graph illustrates the inverse correlation between correct response rate of negative word - face pairs and the contrast estimates during encoding of negative word - face pairs in the region of interest shown in Figure 2A. B. shows the graph illustrates the positive correlation between correct response rate of positive word - face pairs and the contrast estimates during encoding of positive word - face pairs in the region of interest shown in Fig. 2A.

We conducted a secondary correlation analysis to examine the relationship between amygdala activation (mentioned above and shown in Fig. 2A) and the corresponding behavioral performance. This analysis revealed that amygdala activation during encoding of negative word and neutral face pairs (contrast estimate of ‘negative encoding – control’) was inversely correlated (*r* = −0.850, *p* = 0.00006), and that during encoding of positive word and neutral face pairs (contrast estimate of positive encoding – control) was positively correlated (*r* = 0.599, *p* = 0.018) (Fig. 3A) with successful retrieval (Fig. 3B). Amygdala activation during retrieval was not significantly correlated with a correct response rate regardless of whether the words were positive (*r* = 0.274, *p* = 0.322) or negative (*r* = −0.165, *p* = 0.557).

## Discussion

In this study, we explored the effect of emotion on associative memory performance and its underling neural mechanisms. The hippocampus showed activations during the encoding and retrieval of word and face pairs regardless of whether the words were negative or positive. However, there wasn't positive correlation between activations in these regions and associative memory performances, and on the contrary, there was significant negative correlations between left hippocampus activation during negative encoding and the rate of successful retrieval. In contrast, left amygdala activation was observed only during encoding with negative emotionality, and there was also significant negative correlations between this amygdala activation and successful retrieval. In addition, a 2×2 ANOVA and subsequent Post-hoc analysis detected the region activated specifically during encoding with negative emotionality in the left amygdala. Furthermore, this amygdala activation was inversely correlated with subsequent memory retrieval with high significance. These results suggest that amygdala activation induced by negative emotionality may disrupt associative memory encoding.

Although research on memory and emotion has demonstrated that emotional (both positive and negative) events are often better remembered than neutral events [13], [14], we reported previously that negative emotionality does not enhance memory for associated word pairs [12]. In this study, we demonstrated that paired items with negative emotionality are more poorly remembered than those with positive emotionality. In this discrepancy, the encoding of an association between items may have played a role, as compared to encoding single items. This hypothesis is supported by the results of previous studies [21], [22], [24] demonstrating that negative emotion enhances memory for gist, but reduces memory for detail. Although the face and emotional word associative memory assessment in the present study are quite different from the gist and event detail assessments used by previous studies, it is possible that the negative word meanings operate as gist, while the relationships between word and face pairs operate as more peripheral, less salient aspect of the encoding task. This emotion-related effect of memory may be mediated by the amygdala, as suggested by the absence of the effect in individuals with amygdala damage [25]. However, the biological mechanism of such phenomenon has not been examined in detail in human functional neuroimaging studies. The pronounced inverse correlation between amygdala activation induced by negative emotionality and the correct response rate shown in this study provide direct evidence that amygdala activation during encoding is a mediator of this phenomenon. Although we do not know the neural mechanisms responsible for the disruption of associative memory encoding with negative emotionality by amygdala activation, one possible mechanism is that amygdala activation enhances the attention to the negative word itself and reduces the attention to the association of the items required for task performance. This interpretation is consistent with the idea that the amygdala focuses processing resources on the most salient information, as Easterbrook originally proposed [26]. In fact, our regression analyses also revealed a significant inverse correlation between the correct response rate and the magnitude of brain activation in the left hippocampus. This means that activation of this region also disrupted rather than contributed to the associative memory processing. Given the fact that amygdala activity has been reported to correlate with subsequent memory for emotional material [15], [16], [27] and the influence of the amygdala on the efficacy of encoding is believed to be expressed through its effect on the hippocampus, it is plausible that the amygdala may focus processing recourses automatically on the negative words and not the association of paired items required for task performance.

In addition to the results mentioned above, amygdala activation during the positive encoding was positively correlated with the task performance of associative memory in this study. Although the mechanism of this inverse effect of amygdala activation on associative memory is unclear, results of previous studies of patients with amygdalar damage suggest that the amygdala can both potentiate and reduce gist memory, depending on the encoding context [25]. Although additional research is required to better understand the circumstances (positive or negative, single item or paired item) in which amygdala activity can disrupt or facilitate memory encoding, the number of cues to which an organism attends may be modulated by emotional valence and not by arousal mediated by the amygdala.

There are certain limitations in this study that should be taken into consideration. First, we did not include a neutral word condition, so there is the possibility that the significant difference of associative memory performance between negative and positive condition was due to the enhancing effect of the positive word rather than the disrupting effect of the negative word. Although our previous study demonstrated that the correct response rate of negative word pairs was significantly lower than that of neutral word pairs [12], it remains unclear whether the effect of a negative word on face-word associative memory performance would be significant relative to neutral stimuli. However, it is plausible that amygdala activation during negative encoding disrupt the associative memory performance from the pronounced inverse correlation between amygdala activation and associative memory performance. Second, we did not evaluate unpleasantness during the task, so the direct relationship between amygdala activation and negative emotionality is not necessarily clear in this study, although it is reasonable to consider that amygdala activation was induced by negative emotionality of words. Third, as in previous studies [10], [28], our control (baseline) task did not include real faces, so our results of one sample t-test included the regions which were related to the face perception as well as memory and emotion. However, face perception was equally included in each task (negative encoding, positive encoding, negative retrieval, or positive retrieval), and our results of a 2×2 ANOVA was never confounded by face perception. Fourth, we could not examine whether amygdala activation was positively or negatively correlated with the memory for the words themselves, so we could not directly compare the role of amygdala activation between single item memory and associative memory encoding. Further study is needed to address this issue. Finally, we did not use an event-related subsequent memory design which would be appropriate to directory justify the role of brain activation on associative memory performance, but the block-design in which we can raise the BOLD signal to measurable level in shorter scanning run. The major reason of this selection of design was that we were interested in examining the neural activity of associative memory performance in each individual subject, and applying such paradigm to psychiatric disorders like depression that cannot be forced into longer scanning run.

In conclusion, we observed that associative memory encoding is differentially modulated by amygdala activation according to the valence of emotionality. In particular, robust inverse correlation between the amygdala activation during encoding with negative emotionality and associative memory performance was observed. These findings suggest that amygdala activation induced by negative emotion may automatically focus processing resources on the most salient information, and disrupt associative memory encoding directed by instruction.

## Materials and Methods

### Ethics Statement

The study was conducted under a protocol that was approved by the Ethics Committee of Hiroshima University. All subjects submitted informed written consent of their participation.

### Subjects

Fifteen healthy volunteers (6 men and 9 women), aged 21–27 years (mean age ± SD = 23.6±1.9 years), with no history of neurological or psychiatric illness, participated in the study. All subjects showed a similar level of intelligence as assessed by the Japanese Adult Reading Test (JART) (112.5±5.6).

### Experimental task

During the fMRI protocol, subjects performed a novel block-designed face-emotional word paired associate task. We developed this task from a face-name paired associate task [10], [28] that included 3 distinct conditions: encoding, distracter (active baseline), and recognition. The task consisted of 18 blocks, each of which was preceded by an instruction slide informing the subject whether the block was encoding, control, or retrieval condition. Of these 18 blocks, 6 were encoding conditions, 6 were control conditions, and 6 were retrieval conditions. Conditions were interleaved and repeated 6 times (Fig. 1). The duration of each condition was 24 seconds and the preceding instruction slide was shown for 4 seconds. This resulted in a total task period of 9 minutes.

During encoding, pairs of a face and an emotional word were presented serially every 3s, and subjects were asked to remember each face-emotional word pair by pressing a button. The active baseline (control) required subjects to press a button when 2 of 3 words disappeared (randomly within a 3s interval). We used the same emotional words during the corresponding control condition, so as to focus on how emotions modulate the associative memory processing and not on emotional responses themselves. During the retrieval condition, each face was shown with 3 emotional words every 3s, and the subjects were asked to indicate, via button press, which word was previously paired with that face. Forty-eight neutral faces paired with 6 emotional words (3 positive and 3 negative) were used during the experiment, because of the difficulty to select 48 appropriate emotional personality trait words. Although this means that the same words were repeatedly presented with different faces, a different face was presented every time and this task did not require the ability to overcome interference. That is, 48 pairs were presented within encoding condition, and no face-word pair was repeated during the experiment. Each retrieval block tested memory for only the pairs that were in the preceding encoding block, but the presentation of faces was not in the same order in the retrieval block as they were presented in the encoding block. Neutral faces were selected from the database of SOFTPIA JAPAN (The database is not available on line to protect the privacy of subjects participates in the database). Three positive words and 3 negative wards were selected from Anderson's list of personality-trait words translated into Japanese, and were rated on emotional valence and familiarity by a different group of participants [29]. Positive words were from the top 20 positive words and negative words were from the bottom 20 negative words of this list. Positive and negative words were matched in familiarity and word length. Each face and word pair was presented only once during the encoding tasks. For the retrieval tasks performed after the encoding tasks, the remaining 2 of the 3 words were used as distracters. The negative and positive conditions were counterbalanced across the subjects. Stimuli were generated using a personal computer with Presentation software (Neurobehavioral Systems, Inc.; San Francisco, CA). Using an angled mirror, participants viewed the stimuli on a back projection screen mounted outside the scanner bore.

### Acquisition of MRI data

Imaging data were acquired using a GE 3.0 T scanner (General Electric, Milwaukee, Wisconsin). A time course series of 190 volumes per participant (including pre- and post-task period) was acquired with echo planar imaging sequences (TR = 3000 ms, TE = 27 ms, FA = 90deg, Matrix size = 64×64, FOV = 256 mm, 4 mm slice thickness, 32 slice, no gap). Functional scans lasted 9 minutes 30 seconds. After functional scanning, structural scans were acquired using T1-weighted gradient echo pulse sequences (TR = 7.2 ms, TE = 2.1 ms, FA = 20deg, Matrix size = 256×256, FOV = 256 mm, 1 mm slice thickness, 184 slice).

### Analysis

Data were analyzed using the statistical parametric mapping software package, SPM8 (Wellcome Department of Cognitive Neurology, London, UK). The first 5 volumes of the fMRI run (pre-task period) were discarded to ensure a steady-state MR signal, and the remaining 185 volumes were used for the statistical analysis. Each set of functional volumes was realigned to the first volume, spatially normalized to a standard template based upon the Montreal Neurological Institute (MNI) reference brain, and spatially smoothed using an 8-mm FWHM Gaussian kernel.

We modeled four contrasts for each individual, using a general linear model that included each condition (negative encoding, positive encoding, negative retrieval, and positive retrieval) compared to the relevant control conditions. Then, second level analyses were performed according to a random effect model. First, one sample *t*-tests were performed for each contrast. The statistical threshold of *p*<0.05, corrected for whole-brain family wise error (FWE) at a peak level was used, except for *a priori* hypothesized regions, which were thresholded at *p*<0.05, and corrected for small volume (search volume is *a priori* region of interest mask) FWE at a peak level. These *a priori* regions of interest included the hippocampus and amygdala, a region implicated in the processing of memory and emotion. The hippocampal and amygdalic region of the interest mask was created in Montreal Neurological Institute (MNI) space using the WFU Pick Atlas [30]. We used WFU Pick Atlas only for creating the hippocampal and amygdalic region of interest mask, and all other analyses were conducted by using SPM 8.

Second, Pearson's correlation analyses were performed using the averages of contrast estimates (negative encoding-control, positive encoding-control, negative retrieval-control, and positive retrieval-control) of voxels within the clusters detected by the one sample t-test, in order to examine whether activations of these regions during each condition were correlated with corresponding memory performances. Third, a 2×2ANOVA with factors of task (encoding or retrieval) and emotion (negative or positive) was performed. The statistical threshold for this analysis was also set at *p*<0.05, corrected for FWE at a peak level, and small volume correction (SVC) were applied for the hippocampus and amygdala. Finally, Pearson's correlation analyses were performed using the averages of contrast estimates (negative encoding-control, positive encoding-control, negative retrieval-control, and positive retrieval-control) of voxels within the same amygdala cluster detected in the interaction task×emotion interaction shown in Fig. 2, in order to examine whether activations of this region during each condition was correlated with corresponding memory performances.
